# Procedure‐specific morbidity and pathologic assessment after resection of small (≤20 mm) pancreatic neuroendocrine tumors

**DOI:** 10.1111/jne.70243

**Published:** 2026-08-11

**Authors:** Aya Maekawa, Giampaolo Perri, Stefano Partelli, Charles de Ponthaud, Safi Dokmak, Carsten Jäger, Maria Lavinia Luzietti, Alessio Marchetti, Valentina Andreasi, Anna Battistella, Bengi Su Yilmaz, Andrea Sheel, Kunal Rajput, Haiyi Hu, Umberto Cillo, Alain Sauvanet, Helmut Friess, Ryan D. Baron, Sébastien Gaujoux, Massimo Falconi, Giovanni Marchegiani

**Affiliations:** ^1^ Hepato‐Pancreato‐Biliary and Liver Transplant Surgery Unit, Department of Surgical, Oncological and Gastroenterological Sciences (DiSCOG) University of Padua Padua Italy; ^2^ Pancreatic and Transplantation Surgical Unit, IRCCS San Raffaele Hospital, Vita‐Salute University Milan Italy; ^3^ Department of Pancreatic Surgery Pitié‐Salpétrière Hospital, AP‐HP Paris France; ^4^ Department of HPB Surgery and Liver Transplantation Beaujon Hospital, AP‐HP Nord, Université Paris Cité, CRI‐INSERM UMR 1149 Clichy France; ^5^ Department of Surgery School of Medicine and Health, TUM University Hospital, Klinikum Rechts der Isar, Technical University of Munich Munich Germany; ^6^ Department of Pancreatic Surgery Liverpool University Hospitals NHS Foundation Trust Liverpool UK; ^7^ Institute of Global Health Innovation, Department of Surgery and Cancer Imperial College London London UK; ^8^ Department of Gastroenterology Beijing Friendship Hospital, Capital Medical University, National Clinical Research Center for Digestive Disease Beijing China

**Keywords:** central pancreatectomy, enucleation, morbidity, pancreatic neuroendocrine tumors, postoperative pancreatic fistula

## Abstract

Pancreatic neuroendocrine tumors (PanNETs) are increasingly detected, and the optimal extent of resection remains uncertain because perioperative burden and oncologic assessment may differ by procedure. Enucleation (EN) and central pancreatectomy (CP) are often grouped together as parenchyma‐sparing resections (PSRs) despite substantial differences in technical complexity, postoperative risk, and oncologic evaluation.We conducted a multicenter retrospective study of consecutive patients who underwent resection for pathologically confirmed PanNETs ≤20 mm. Procedures were categorized as EN, CP, pancreatoduodenectomy (PD), total pancreatectomy (TP), or distal pancreatectomy (DP). Primary outcomes were clinically relevant postoperative pancreatic fistula (CR‐POPF) and major morbidity, defined as Clavien‐Dindo grade ≥IIIa. A prespecified pairwise comparison focused on EN and CP, and pathologic assessment patterns were analyzed descriptively across procedures. Of 460 patients, 164 (35.7%) underwent EN, 40 (8.7%) CP, 84 (18.2%) PD/TP, and 172 (37.4%) DP. CR‐POPF occurred in 115 patients (25.0%), major morbidity in 68 (14.8%), and 90‐day mortality in 4 (0.9%). Among PSRs, CP had higher major morbidity than EN (25.0% vs 10.4%, *p* =.020), higher CR‐POPF (50.0% vs. 24.4%, *p* =.003), and a longer median hospital stay (19.5 vs. 10.0 days, *p* <.001). Lymph node (LN) assessment was less frequent after EN than CP (40.4% vs. 87.5%, *p* <.001). Among assessed cases, LN metastases were identified in 44/320 patients (13.8%). EN and CP are not interchangeable as parenchyma‐sparing options for small PanNETs. Among patients selected for resection, procedure selection should consider tumor location, function, technical feasibility, and oncologic requirements rather than size alone.

## Graphical abstract

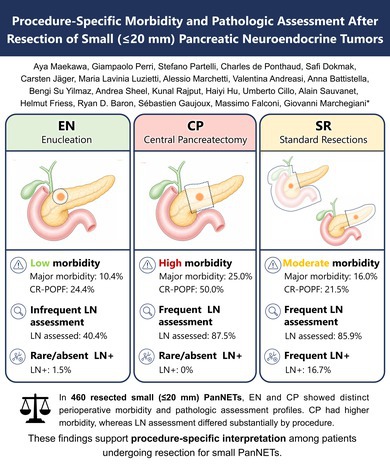

## 1 INTRODUCTION

Small pancreatic neuroendocrine tumors (PanNETs) are increasingly identified, often incidentally, and frequently demonstrate indolent behavior.^1^, ^2^, ^3^ While active surveillance is an established approach for selected small (≤20 mm), asymptomatic nonfunctional PanNETs, a substantial proportion of patients continue to undergo surgical resection due to uncertainties in preoperative risk stratification, as well as anatomical, oncological, or patient‐related factors.^4^, ^5^, ^6^, ^7^ Consequently, clinically actionable evidence should clarify procedure‐specific morbidity alongside oncologic outcomes in order to better guide operative planning.

Parenchyma‐sparing resections (PSR), including enucleation (EN) and central pancreatectomy (CP), were developed to preserve pancreatic endocrine and exocrine function, particularly in patients with small and biologically low‐risk PanNETs.^8^, ^9^, ^10^, ^11^ However, these procedures may carry a unique risk profile, particularly a higher incidence of clinically relevant postoperative pancreatic fistula (CR‐POPF).^12^, ^13^, ^14^ Furthermore, the oncologic adequacy of PSR remains controversial, given the less systematic lymph node (LN) assessment and potential differences in margin evaluation compared to standard pancreatic resections.^15^, ^16^, ^17^ These concerns are relevant even for small tumors, as size alone does not fully indicate biological aggressiveness.

Direct comparisons between PSR and standard resection (SR) are limited by selection bias, as PSR is typically reserved for anatomically suitable, biologically low‐risk lesions, whereas SRs are more often performed for higher‐risk or technically challenging cases.^12^, ^18^ Grouping EN and CP within a single PSR category may also obscure relevant clinical differences in perioperative risk, since these procedures vary substantially in technical complexity, reconstruction requirements, and fistula rates.

Therefore, multicenter evidence focusing on procedure‐specific morbidity and pathologic assessment is needed to better inform operative planning for small PanNETs, particularly to clarify differences between EN, CP, and standard pancreatic resections. We aimed to compare the procedure‐specific perioperative burden of EN and CP, and to describe patterns of pathologic and LN assessment across procedures among patients selected for resection.

## 2 MATERIALS AND METHODS

### 2.1 Study design and population

**FIGURE 1:**
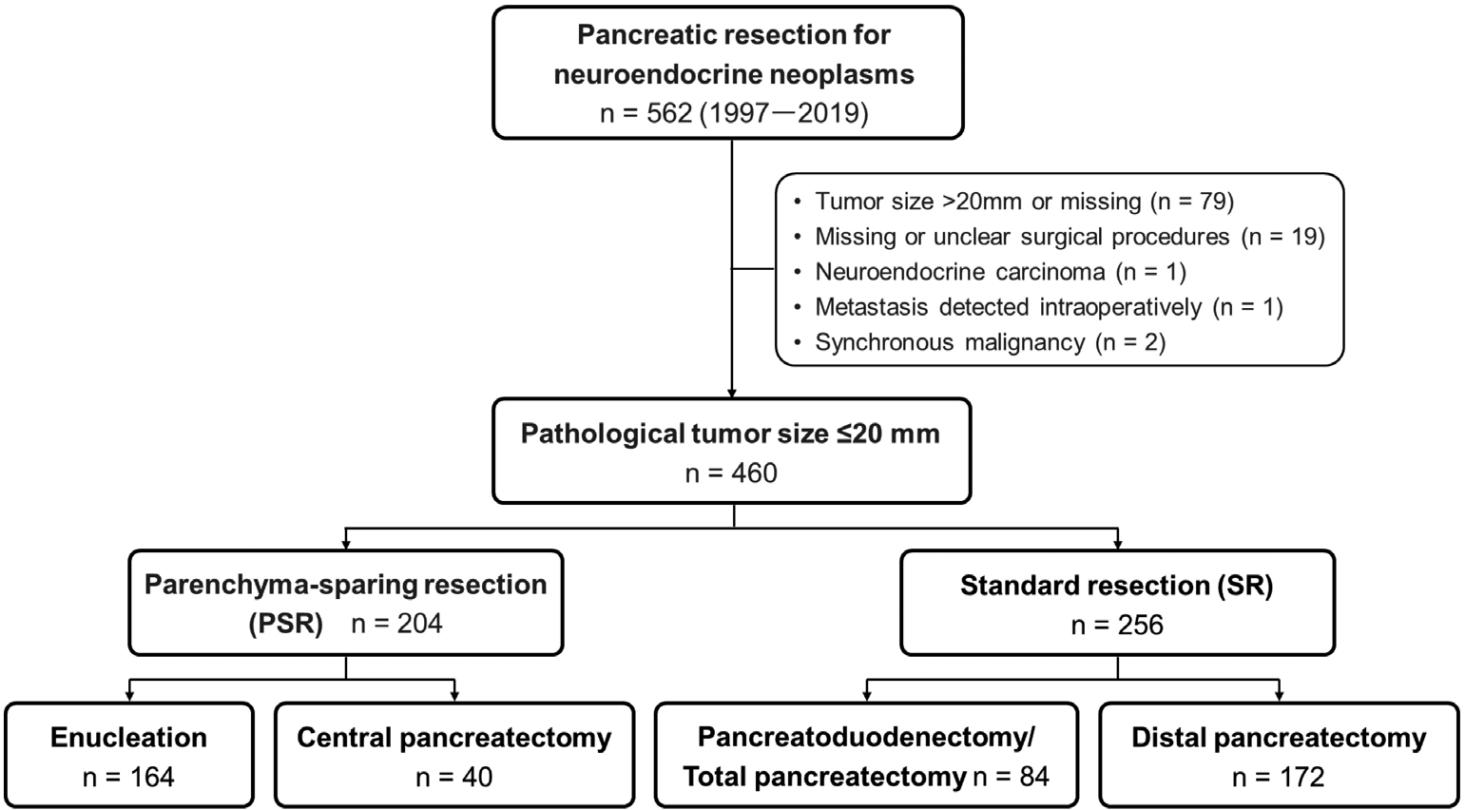
Study design.

A multicenter retrospective cohort study was conducted, including consecutive patients who underwent surgical resection for primary PanNETs at five international institutions from 1997 to 2019. Data were extracted from institutional databases and electronic medical records using a standardized case report form. The study was approved by the institutional review board or ethics committee at each participating institution, with informed consent waived as applicable for retrospective research (Figure 1).

Eligible patients underwent surgical resection with pathologically confirmed PanNETs and had available perioperative outcome data. Exclusion criteria included neuroendocrine carcinoma, non‐pancreatic primary tumors or metastatic NET involving the pancreas, missing pathological tumor size, indeterminate operative details precluding classification of resection type, and missing or inconsistent perioperative outcome data.

### 2.2 Definitions

#### 2.2.1 Resection types and tumor characteristics

Resection types were categorized as EN, CP, pancreatoduodenectomy or total pancreatectomy (PD/TP), and distal pancreatectomy (DP). PSR included EN and CP, while SR comprised PD/TP and DP. PD/TP and DP were reported separately due to their distinct perioperative risk profiles.

Small PanNETs were defined as tumors with a pathological maximum tumor size ≤20 mm. Tumor grade was defined according to the World Health Organization classification based on the Ki‐67 proliferation index when available, and categorized as G1 (Ki‐67 <3%), G2 (Ki‐67 3–20%), or G3 (Ki‐67 >20%).^19^, ^20^ Patients with missing Ki‐67 data were included and treated as missing in grade‐related analyses.

#### 2.2.2 Postoperative outcomes

Postoperative outcomes were assessed up to 90 days after surgery. Primary postoperative outcomes were CR‐POPF, defined as Grade B or C according to the International Study Group of Pancreatic Surgery (ISGPS) criteria,^21^ and major morbidity, defined as any postoperative complication of Clavien–Dindo grade ≥IIIa.^22^ Other pancreas‐specific complications, including postpancreatectomy hemorrhage (PPH), delayed gastric emptying (DGE), and chyle leak, were defined according to the ISGPS criteria.^23^, ^24^, ^25^

Reoperation was defined as any unplanned return to the operating room requiring surgical intervention, while mortality was defined as death from any cause occurring within 90 days after surgery.

#### 2.2.3 Pathologic and oncologic assessment variables

Pathologic variables relevant to oncologic interpretation included tumor size, tumor grade, LN assessment status, LN positivity among assessed cases, and resection margin status. LN assessment was defined as the examination of at least one LN. Within the PSR cohort, patients were further categorized based on whether LN assessment was performed or omitted. Patients with missing information on LN assessment were excluded from this comparison.

Detailed indications for surgery, preoperative imaging findings, granular anatomical eligibility for EN or CP, long‐term pancreatic function, quality of life, and patient‐reported outcomes were inconsistent and therefore excluded from analysis.

### 2.3 Statistical analysis

Baseline characteristics were summarized by resection group. Continuous variables were summarized as median (interquartile range [IQR]), compared using the Kruskal–Wallis test across procedure groups and the Mann–Whitney *U* test for the prespecified pairwise comparison of EN and CP, while categorical variables were compared using Fisher's exact test. Percentages were calculated using available‐case denominators.

The primary analysis focused on comparisons of the primary postoperative outcomes, CR‐POPF and major morbidity, across EN, CP, PD/TP, and DP, with a prespecified pairwise comparison of EN and CP within the PSR cohort. Other postoperative outcomes and pathologic assessment patterns across procedures were analyzed descriptively. To further characterize selection within PSR, patient, tumor, procedural, and selected pathological characteristics were compared between PSR cases with and without LN assessment. Recurrence and death events were summarized descriptively without time‐to‐event analysis.

A two‐sided *p*‐value <.05 was considered statistically significant. Analyses were performed using EZR (Saitama Medical Center, Jichi Medical University), a graphical user interface for R (The R Foundation for Statistical Computing, Vienna, Austria, version 4.2.5).^26^

## 3 RESULTS

### 3.1 Population and surgical procedures

A total of 460 patients with small PanNETs were included. The median age was 56.2 years (IQR 46.2–65.9), and 51.7% were male. Additional baseline characteristics are summarized in Table 1.

**TABLE 1:** Baseline characteristics and operative details of small pancreatic neuroendocrine neoplasms (≤20 mm).

Characteristics	Total (*n* = 460)	EN (*n* = 164, 35.7%)	CP (*n* = 40, 8.7%)	PD/TP (*n* = 84, 18.2%)	DP (*n* = 172, 37.4%)	*p*‐value (overall)	*p*‐value (EN vs. CP)
Baseline							
Age, median (IQR), years	56.2 (46.2–65.9)	52.4 (42.9–63.1)	50.5 (43.0–60.1)	56.2 (47.2–65.3)	60.6 (51.3–68.2)	**<.001**	.594
Male sex, no. (%)	238 (51.7)	83 (50.6)	18 (45.0)	49 (58.3)	88 (51.2)	.519	.598
BMI, median (IQR), kg/m^2^	25.1 (22.7–28.7)	24.7 (22.3–28.9)	25.0 (23.3–28.1)	25.1 (22.5–27.3)	25.8 (23.2–29.4)	.467	.550
Diabetes mellitus, no. (%)	47 (10.2)	9 (5.8)	6 (15.4)	11 (13.9)	21 (14.0)	.**049**	.085
Tumor location, no. (%)						**<.001**	**<.001**
Head	138 (30.0)	66 (40.2)	1 (2.5)	71 (84.5)	0 (0.0)		
Body	193 (42.0)	66 (40.2)	39 (97.5)	3 (3.6)	85 (49.4)		
Tail	116 (25.2)	30 (18.3)	0 (0.0)	3 (3.6)	83 (48.3)		
Multiple	13 (2.8)	2 (1.2)	0 (0.0)	7 (8.3)	4 (2.3)		
Tumor functionality, no. (%)						**<.001**	**<.001**
Non‐functional	294 (63.9)	82 (50.0)	32 (80.0)	60 (71.4)	120 (69.8)		
Insulinoma	148 (32.2)	79 (48.2)	7 (17.5)	17 (20.2)	45 (26.2)		
Other functional	18 (3.9)	3 (1.8)	1 (2.5)	7 (8.3)	7 (4.1)		
Intraoperative details							
Minimally invasive surgery, no. (%)	155 (33.7)	48 (29.3)	14 (35.0)	10 (11.9)	83 (48.3)	**<.001**	.576
Blood loss, median (IQR), mL	100.0 (50.0–200.0)	100.0 (50.0–150.0)	200.0 (75.0–200.0)	300.0 (187.5–500.0)	160.0 (81.3–400.0)	**<.001**	.**016**

*Note*: Percentages were calculated using available‐case denominators.

Abbreviations: BMI, body mass index; CP, central pancreatectomy; DP, distal pancreatectomy; EN, enucleation; IQR, interquartile range; PD, pancreatoduodenectomy; TP, total pancreatectomy.

Resection types comprised EN (*n* = 164, 35.7%), CP (*n* = 40, 8.7%), PD/TP (*n* = 84, 18.2%), and DP (*n* = 172, 37.4%). Functional tumors were more common in EN than CP, reflecting a higher proportion of insulinomas in the EN group (48.2% vs. 17.5%, EN vs. CP *p* < .001). Non‐insulinoma functional tumors were uncommon and heterogeneous, consisting mainly of gastrinomas (*n* = 6), with only isolated cases of glucagonoma, vasoactive intestinal peptide‐secreting tumor (VIPoma), somatostatinomas, adrenocorticotropic hormone‐secreting tumor (ACTHoma), and serotonin‐producing tumor. Minimally invasive surgery was performed in 155 patients overall (33.7%), including 48/164 (29.3%) after EN and 14/40 (35.0%) after CP (EN vs. CP *p* = .576) (Table 1).

### 3.2 Postoperative outcomes

In the overall cohort, CR‐POPF occurred in 115 patients (25.0%), major morbidity in 68 (14.8%), and 90‐day mortality in 4 (0.9%). The median length of hospital stay (LOS) was 11.0 days (IQR 8.0–17.0) (Tables 2 and S1).

**TABLE 2:** Postoperative and pathological outcomes of small pancreatic neuroendocrine neoplasms (≤20 mm).

Characteristics	Total (*n* = 460)	EN (*n* = 164)	CP (*n* = 40)	PD/TP (*n* = 84)	DP (*n* = 172)	*p*‐value (overall)	*p*‐value (EN vs. CP)
Postoperative outcomes							
CR‐POPF, no. (%)	115 (25.0)	40 (24.4)	20 (50.0)	18 (21.4)	37 (21.5)	.**004**	.**003**
PPH, no. (%)	27 (5.9)	8 (4.9)	6 (15.0)	7 (8.3)	6 (3.5)	.**035**	.**035**
DGE, no. (%)	54 (11.7)	17 (10.4)	16 (40.0)	14 (16.9)	7 (4.1)	**<.001**	**<.001**
Major morbidity, no. (%)	68 (14.8)	17 (10.4)	10 (25.0)	19 (22.6)	22 (12.8)	.**016**	.**020**
LOS, median (IQR), days	11.0 (8.0–17.0)	10.0 (8.0–14.0)	19.5 (14.0–31.0)	14.0 (10.0–21.0)	10.0 (8.0–14.0)	**<.001**	**<.001**
Pathology							
Tumor size, median (IQR), mm	14.0 (10.0–17.0)	14.0 (10.0–17.0)	15.0 (9.0–17.3)	14.0 (10.0–16.3)	15.0 (10.0–17.0)	.624	.432
Ki‐67, median (IQR), %	1.0 (1.0–2.0)	1.0 (1.0–2.0)	1.0 (1.0–2.0)	1.0 (1.0–2.0)	1.0 (1.0–2.0)	.459	.408
Tumor grade						.175	.509
G1	362 (78.7)	130 (79.3)	34 (85.0)	67 (79.8)	131 (76.2)		
G2	89 (19.3)	34 (20.7)	6 (15.0)	14 (16.7)	35 (20.3)		
Missing	9 (2.0)	0 (0.0)	0 (0.0)	3 (3.6)	6 (3.5)		
LN assessment^a^ (≥1 LN), no. (%)	320 (69.6)	65 (40.4)	35 (87.5)	83 (98.8)	137 (81.5)	**<.001**	**<.001**
Positive LN status, no. (%)	44 (13.8)	1 (1.5)	0 (0.0)	28 (33.7)	15 (10.9)	**<.001**	1.000
R0 status, no. (%)	422 (93.2)	142 (88.8)	40 (100.0)	81 (96.4)	159 (94.1)	.**026**	.**027**

*Note*: Percentages were calculated using available‐case denominators.

Abbreviations: CP, central pancreatectomy; CR, clinically relevant; DGE, delayed gastric emptying; DP, distal pancreatectomy; EN, enucleation; IQR, interquartile range; LN, lymph node; LOS, length of hospital stay; PD, pancreatoduodenectomy; POPF, postoperative pancreatic fistula; PPH, postpancreatectomy hemorrhage; TP, total pancreatectomy.

^a^ For LN‐related variables, percentages were calculated among patients with LN assessment (≥1 LN examined).

Within PSR, CP was associated with significantly higher postoperative morbidity than EN. Major morbidity occurred in 10/40 (25.0%) of CP cases compared to 17/164 (10.4%) of EN cases (*p* = .020). CR‐POPF was observed in 20/40 (50.0%) after CP and 40/164 (24.4%) after EN (*p* = .003). CP also had higher rates of DGE (16/40 [40.0%] vs. 17/164 [10.4%], *p* < .001), PPH (6/40 [15.0%] vs. 8/164 [4.9%], *p* = .035), and a longer LOS (19.5 vs. 10.0 days, *p* < .001) (Table 2).

### 3.3 Pathologic findings and oncologic assessment patterns

The median tumor size was 14.0 mm (IQR 10.0–17.0). Most tumors were G1 (362/460 [78.7%]), with 19.3% classified as G2 (Table 2).

Pathologic assessment patterns varied by surgical procedure. LN assessment (≥1 LN examined) was performed in 320/460 patients (69.6%), and was markedly lower in EN than CP (65/164 [40.4%] vs. 35/40 [87.5%], *p* < .001). Among those assessed, LN metastases were identified in 44/320 patients (13.8%), and were more frequent in G2 than G1 tumors (14/63 [22.2%] vs. 28/252 [11.1%], *p* = .036). LN positivity was highest after PD/TP (28/83, 33.7%), followed by DP (15/137, 10.9%), with only one positive case after EN (1/65, 1.5%) and none (0/35) after CP (Table 2 and Figure S1). R0 resection was achieved in 422/453 (93.2%) of evaluable patients and differed across procedures, including between EN and CP (88.8% vs. 100.0%, EN vs. CP *p* = .027) (Table 2).

### 3.4 Characteristics of PSR according to LN assessment status

Within the PSR cohort, LN assessment was performed in 100 patients and omitted in 101; three patients with missing data were excluded (Table 3). Patients undergoing PSR with LN assessment were younger than those who did not (median 50.3 vs. 55.0 years, *p* = .036). Non‐functional tumors were more common among those with LN assessment (69.0% vs. 42.6%), whereas insulinomas were more common when LN assessment was omitted (29.0% vs. 55.4%, *p* < .001).

**TABLE 3:** Characteristics of parenchyma‐sparing resections according to lymph node assessment status.

Characteristics	LN assessed (*n* = 100)	No LN assessment (*n* = 101)	*p*
Patient and tumor characteristics			
Age, median (IQR), years	50.3 (42.9–57.8)	55.0 (42.8–66.8)	.**036**
Male sex, no. (%)	47 (47.0)	53 (52.5)	.482
BMI, median (IQR), kg/m^2^	24.8 (22.3–27.9)	24.9 (22.8–29.1)	.557
Diabetes mellitus, no. (%)	8 (8.4)	6 (6.3)	.782
Tumor location, no. (%)			.179
Head	31 (31.0)	34 (33.7)	
Body	56 (56.0)	48 (47.5)	
Tail	11 (11.0)	19 (18.8)	
Multiple	2 (2.0)	0 (0.0)	
Tumor functionality, no. (%)			**<.001**
Non‐functional	69 (69.0)	43 (42.6)	
Insulinoma	29 (29.0)	56 (55.4)	
Other functional	2 (2.0)	2 (2.0)	
Procedure			
Surgical type, no. (%)			**<.001**
Enucleation	65 (65.0)	96 (95.0)	
Central pancreatectomy	35 (35.0)	5 (5.0)	
Pathologic characteristics			
Tumor size, median (IQR), mm	15.0 (10.0–17.0)	14.0 (10.0–17.0)	.817
Tumor grade			.285
G1	84 (84.0)	78 (77.2)	
G2	16 (16.0)	23 (22.8)	
R0 status, no. (%)	94 (95.9)	87 (87.0)	.**040**

*Note*: Percentages were calculated using available‐case denominators.

Abbreviations: BMI, body mass index; IQR, interquartile range; LN, lymph node.

The distribution of PSR procedure types differed substantially by LN assessment status. Among PSR cases with LN assessment, EN was performed in 65/100 (65.0%), compared with 96/101 (95.0%) without LN assessment (*p* < .001), whereas CP was more frequent when LN assessment was performed. Tumor size and grade did not differ significantly according to LN assessment status (Table 3).

### 3.5 Recurrence and survival status

Recurrence status was available for 460 patients, with a median follow‐up of 50.7 months (IQR 19.3–92.7). Recurrence occurred in 27 patients overall (5.9%) (5 after EN, 2 after CP, and 20 after SR; overall *p* = .201). At the last follow‐up, 23 deaths were recorded (EN: 3, 1.8%; CP: 0, 0%; SR: 20, 7.8%), including three disease‐related deaths in patients with distant metastases. Given the low number of recurrence and disease‐related death events, these outcomes were summarized descriptively.

## 4 DISCUSSION

In this multicenter cohort of resected small PanNETs, perioperative burden and pathologic assessment patterns varied significantly by surgical procedure. These results indicate that a simple PSR‐versus‐SR framework does not fully capture the complexity of small PanNET surgery, as the balance between short‐term morbidity and pathologic assessment differs by procedure. This distinction was particularly notable within PSR, where EN and CP have distinct clinical profiles.

Within PSR, CP consistently showed a less favorable short‐term profile than EN. Although CP preserves pancreatic parenchyma, it was associated with a substantial perioperative burden in our cohort. CP should not be considered safer than DP solely because it is parenchyma‐sparing. This difference extended beyond CR‐POPF to other pancreas‐related postoperative events, including DGE and PPH, and was reflected in longer LOS. This pattern is clinically plausible given the technical complexity of CP, which involves parenchymal preservation, pancreatic transection, and reconstruction with two possible sites of pancreas leak. CP remains an important option for selected lesions where EN is not technically appropriate or may be oncologically insufficient, but its use should be weighed carefully against its greater perioperative burden. In highly selected patients requiring more extensive resection, function‐preserving strategies such as islet autotransplantation have been explored in specialized centers,^27^ though not assessed here. In contrast, EN offers a more favorable short‐term balance when anatomically feasible and oncologic concerns are limited.

From a pathologic standpoint, these findings support previous concerns that tumor size alone may not reliably identify a negligible‐risk subgroup among small PanNETs. Although all tumors were ≤ 20 mm, a substantial proportion was G2, and LN metastases were found in node‐assessed patients. This suggests that small size does not always indicate low biological risk. Oncologic interpretation should be cautious, as LN assessment was less frequent after EN than after CP or SR. Differences in LN positivity likely reflect both tumor biology and procedure‐dependent assessment, as well as pathology practices. The prognostic significance of LN positivity in small PanNETs also remains unclear, as LN involvement may influence staging more than long‐term survival.^28^, ^29^, ^30^ Similarly, margin status differences should not be considered definitive evidence of oncologic adequacy without robust time‐to‐event outcomes.^31^

The analysis within PSR further supports this interpretation. LN assessment was more common in non‐functional tumors and in cases treated with CP, while insulinomas and EN were more frequent among PSR cases without LN assessment. Tumor size and grade did not differ significantly between groups. These findings suggest that, in practice, LN evaluation within PSR was guided more by clinical judgment, including tumor functionality, operative strategy, and perceived oncologic risk, rather than by size alone. The predominance of insulinoma among cases without LN assessment reflects its favorable biology and symptom‐driven surgical indication.^32^, ^33^

These findings should be viewed in the context of the current trend toward more selective surgery for small pancreatic lesions. A conceptually similar shift has occurred in managing small pancreatic cysts and branch‐duct intraductal papillary mucinous neoplasms, where surveillance has become standard for lesions without high‐risk features.^34^, ^35^, ^36^, ^37^ Current evidence supports active surveillance for selected asymptomatic sporadic nonfunctioning PanNETs ≤20 mm without aggressive features.^5^, ^20^ The key message is not to broaden surgical indications, but to clarify that EN and CP are not interchangeable when surgery is chosen. The present study should not be interpreted as defining which small PanNETs should undergo resection instead of surveillance, as detailed surgical indications such as tumor growth, symptom burden, radiologic findings, patient preference, and multidisciplinary judgment were not consistently recorded. For anatomically suitable lesions with limited oncologic concern, EN offers a more favorable postoperative profile than CP.

Another important consideration is the potential role of minimally invasive, particularly robotic, approaches in parenchyma‐sparing pancreatic surgery.^38^, ^39^, ^40^, ^41^, ^42^ These techniques may improve short‐term outcomes, especially in technically demanding procedures such as CP, although robust procedure‐specific evidence remains limited.

This study has several limitations. The retrospective design introduces unavoidable heterogeneity in clinical practice, pathologic assessment, and follow‐up across institutions and time periods. Comprehensive data on surgical indications, such as tumor growth during surveillance, symptoms, radiologic findings, patient preference, and multidisciplinary input, were not consistently available. While tumor location was recorded, detailed anatomical information, including neck or proximal body location, proximity to the main pancreatic duct, and technical suitability for EN or CP, was not systematically collected. Oncologic interpretation is also limited by procedure‐dependent differences in LN assessment. Preoperative imaging, including assessment of suspicious LNs, was also inconsistent, and radiologic evaluation may not detect microscopic nodal disease. Importantly, we lacked detailed data on long‐term pancreatic function in both endocrine and exocrine insufficiency, as well as patient‐reported outcomes, which are central to the rationale for PSR, especially CP. Therefore, although CP showed a substantial perioperative burden, we could not determine whether this short‐term disadvantage may be offset by a meaningful long‐term functional benefit in selected patients. The small number of recurrence and death events, along with heterogeneity in follow‐up, limited robust time‐to‐event analyses. Future studies with standardized reporting are needed to determine when the tradeoff associated with CP is justified from a patient‐centered perspective.

In conclusion, among patients selected for resection, EN and CP are not equivalent forms of PSR in small PanNETs. CP should not be viewed as a lower‐risk procedure solely because it preserves pancreatic parenchyma. When surgery has been selected, procedure selection should be individualized, considering tumor function, lesion location, technical feasibility, perioperative risk, and oncologic requirements rather than based on a single‐size threshold.

## AUTHOR CONTRIBUTIONS

**Haiyi Hu:** Investigation; writing – review and editing. **Stefano Partelli:** Investigation; writing – review and editing. **Safi Dokmak:** Investigation; writing – review and editing. **Valentina Andreasi:** Investigation; writing – review and editing. **Anna Battistella:** Investigation; writing – review and editing. **Aya Maekawa:** Writing – original draft; formal analysis; data curation; conceptualization; methodology; visualization; project administration. **Andrea Sheel:** Investigation; writing – review and editing. **Charles de Ponthaud:** Investigation; writing – review and editing. **Kunal Rajput:** Investigation; writing – review and editing. **Giampaolo Perri:** Conceptualization; methodology; formal analysis; writing – review and editing; supervision; visualization; project administration. **Alessio Marchetti:** Methodology; writing – original draft. **Bengi Su Yilmaz:** Investigation; writing – review and editing. **Maria Lavinia Luzietti:** Investigation; writing – review and editing. **Massimo Falconi:** Investigation; writing – review and editing. **Ryan D. Baron:** Investigation; writing – review and editing. **Umberto Cillo:** Investigation; writing – review and editing. **Sébastien Gaujoux:** Investigation; writing – review and editing. **Alain Sauvanet:** Investigation; writing – review and editing. **Helmut Friess:** Investigation; writing – review and editing. **Carsten Jäger:** Investigation; writing – review and editing. **Giovanni Marchegiani:** Writing – review and editing; conceptualization; supervision; project administration; methodology.

## FUNDING INFORMATION

This research received no specific grant from any funding agency in the public, commercial, or not‐for‐profit sectors.

## CONFLICT OF INTEREST STATEMENT

Dr. Ryan D. Baron received an honorarium from Novartis Pharmaceuticals UK Ltd. for a guest lecture outside the submitted work. All other authors declare no conflicts of interest.

## ETHICS STATEMENT

This multicenter retrospective study was approved by the institutional review board or ethics committee at each participating institution.

## PATIENT CONSENT STATEMENT

The requirement for informed consent was waived, as applicable, owing to the retrospective nature of the study.

## Supporting information


**Figure S1.** Lymph node assessment and nodal status by resection type in small pancreatic neuroendocrine tumors (≤20 mm). Bars represent the proportions of patients without lymph node (LN) assessment, with LN‐assessed/LN‐negative status, and with LN‐assessed/LN‐positive status for each resection group. Percentages are based on the total number of patients per group. CP, central pancreatectomy; DP, distal pancreatectomy; EN, enucleation; PD, pancreatoduodenectomy; TP, total pancreatectomy.

**Table S1.** Additional perioperative variables and postoperative outcomes of small pancreatic neuroendocrine neoplasms (≤20 mm).

## Data Availability

The datasets generated and analyzed during the current study are not publicly available, as consent was not provided by the participants to share their data with third parties.
