# Supplementary material for: Procedure‐specific morbidity and pathologic assessment after resection of small (≤20 mm) pancreatic neuroendocrine tumors
Source: J Neuroendocrinol. 2026 Aug 11;38(8):e70243. doi: 10.1111/jne.70243 (PMC13461252; doi:10.1111/jne.70243)
Supplement: Supplementary file 1 [file JNE-38-e70243-s002.docx]

| **Table S1. Additional perioperative variables and postoperative outcomes of small pancreatic neuroendocrine neoplasms (≤20 mm)** | | | | | | | |
| --- | --- | --- | --- | --- | --- | --- | --- |
| **Characteristics** | **Total**  **(n = 460)** | **EN**  **(n = 164)** | **CP**  **(n = 40)** | **PD/TP**  **(n = 84)** | **DP**  **(n = 172)** | **P value**  **(Overall)** | **P value**  **(EN vs CP)** |
| ASA score ≥3, No. (%) | 31 (6.7) | 13 (20.0) | 3 (10.3) | 6 (13.3) | 9 (17.0) | 0.696 | 0.375 |
| Venous resection, No. (%) | 3 (0.7) | 1 (0.6) | 0 (0.0) | 1 (1.2) | 1 (0.6) | 0.849 | 1.000 |
| POPF, No. (%) | 136 (29.6) | 53 (32.3) | 25 (62.5) | 20 (23.8) | 38 (22.1) | **<0.001** | **<0.001** |
| Biliary fistula, No. (%) | 12 (2.6) | 3 (1.8) | 2 (5.0) | 6 (7.2) | 1 (0.6) | **0.010** | 0.254 |
| Reoperation, No. (%) | 23 (5.0) | 8 (4.9) | 4 (10.0) | 5 (6.0) | 6 (3.5) | 0.327 | 0.257 |
| Clavien-Dindo, No. (%) |  |  |  |  |  |  | **0.001** |
| 0 | 161 (35.0) | 56 (34.1) | 5 (12.5) | 43 (51.2) | 57 (33.1) |  |  |
| 1 | 95 (20.7) | 49 (29.9) | 7 (17.5) | 6 (7.1) | 33 (19.2) |  |  |
| 2 | 136 (29.6) | 42 (25.6) | 18 (45.0) | 16 (19.0) | 60 (34.9) |  |  |
| 3 | 57 (12.4) | 12 (7.3) | 9 (22.5) | 15 (17.9) | 21 (12.2) |  |  |
| 4 | 8 (1.7) | 5 (3.0) | 1 (2.5) | 1 (1.2) | 1 (0.6) |  |  |
| 5 | 3 (0.7) | 0 (0.0) | 0 (0.0) | 3 (3.6) | 0 (0.0) |  |  |
| 90-day mortality, No. (%) | 4 (0.9) | 0 (0.0) | 0 (0.0) | 4 (4.8) | 0 (0.0) | **0.003** | 1.000 |
| *Abbreviations: EN, enucleation; CP, central pancreatectomy; PD, pancreatoduodenectomy; TP, total pancreatectomy; DP, distal pancreatectomy; IQR, interquartile range; ASA, American Society of Anesthesiologists Classification; POPF, postoperative pancreatic fistula.*  *Percentages were calculated using available-case denominators.* | | | | | | | |
